# Global, Regional, and National Burden of Aortic Aneurysm and Its Attributable Risk Factors from 1990 to 2021: An Analysis of the Global Burden of Disease Study 2021

**DOI:** 10.34172/aim.34264

**Published:** 2025-07-01

**Authors:** Huanan Liu, Xiaoshen Zhang, Hua Lu

**Affiliations:** ^1^Department of Cardiovascular Surgery, The First Affiliated Hospital of Jinan University, Guangzhou 510630, China

**Keywords:** Aortic aneurysm, Deaths, Disability-adjusted life years, Global Burden of Disease, Risk factors

## Abstract

**Background:**

Aortic aneurysm (AA) remains a significant global cause of mortality. This study aimed at systematically revealing the distribution of AA burden and its attributable risk factors from 1990 to 2021.

**Methods:**

Data of AA-related deaths, disability-adjusted life years (DALYs), and corresponding age-standardized rates (ASRs) were extracted from the Global Burden of Disease (GBD) 2021 study. Estimated annual percentage change (EAPC) was calculated to assess trends in the AA burden at global, regional, and national levels. The temporal trends of AA burden were analyzed, and key attributable risk factors were identified. Population attributable fractions (PAFs) were calculated to assess the impact of these risk factors.

**Results:**

From 1990 to 2021, global AA-related deaths increased by 74.218% (95% UI: 83090‒93492 to 138413‒165739), while age-standardized death rates (ASDR) declined by 26.772% (95% UI: 2.538 to 1.861 per 100000). DALYs rose by 64.944% (95% UI: 1784177‒2006704 to 2857320‒3353858), with ASDALYR falling by 25.1% (95% UI: 48.789 to 36.543 per 100000). Regions with high or middle-high socio-demographic index (SDI) scores experienced higher AA burdens compared to lower SDI regions. Males had higher burdens, peaking at ages 70‒74 (deaths) and 65‒69 (DALYs). In regions with an SDI above 0.75, the ASRs of AA burden exhibited a downward trend. Smoking was identified as the most significant attributable risk factor.

**Conclusion:**

From 1990 to 2021, declining trends were observed in the ASRs of AA-related deaths and DALYs, although males reported a higher AA burden than females. Efforts to control tobacco use should be prioritized as a key preventive strategy.

## Introduction

 Aortic aneurysm (AA) is a vascular condition characterized by segmental dilatation of the aorta exceeding 50% of its normal diameter.^1^ It ranks as the fifth leading cause of cardiovascular disease (CVD) disability-adjusted life years (DALYs) in the high-income Asia Pacific region.^2^ and the eighth leading cause of CVD DALYs rates globally.^3^ Among individuals aged over 55 years, AA is the fifteenth leading cause of death.^4^ When associated with atherosclerosis, AA becomes the second most prevalent vascular condition.^5^ AA typically develops without noticeable symptoms but poses a high risk of catastrophic events, including dissections and ruptures, which can lead to sudden death with a survival rate less than 20%.^6^ Compounding this threat, the global burden of key modifiable AA risk factors (notably hypertension, smoking, and atherosclerotic CVD) has been increasing persistently.^7,8^ This confluence of factors necessitates a comprehensive understanding of AA’s evolving epidemiological characteristics to inform effective early screening strategies and timely interventions.

 The Global Burden of Disease (GBD) study serves as a vital ongoing international collaboration, systematically quantifying the global and regional burden of 371 diseases and injuries, alongside 88 risk factors, across 204 countries and territories from 1990 onwards.^9,10^ By synthesizing information from the published literature, surveys, and epidemiological data, the GBD study supports national health authorities in allocating medical resources efficiently. AA was formally integrated into the GBD cause-of-death database in 2010, enabling standardized global tracking.^11^ While previous studies have described aspects of the AA burden using GBD 2019 data, significant limitations inherent in that iteration constrain their current relevance and necessitate an updated analysis.^12,13^ Critically, GBD 2019 lacked data reflecting the profound disruptions of the COVID-19 pandemic on healthcare systems globally. Pandemic-related delays in routine surveillance and elective repairs for AA likely exacerbated outcomes, potentially increasing rupture risks and mortality due to deferred care—trends entirely absent from pre-2020 data.^14^ Furthermore, GBD 2021 includes expanded vital registration (30 + new countries), refined cause-of-death modeling, particularly through updated redistribution algorithms for misclassified CVD deaths (e.g. AA miscoded as “unspecified stroke” or heart failure), and integrates specific adjustments for the pandemic’s impact on mortality data collection and disease burden.^10^ These crucial advancements address critical coverage gaps and estimation biases present in GBD 2019, enabling more reliable cross-national and temporal comparisons.

 Beyond methodological advancements, compelling epidemiological shifts underscore the urgency of re-examining AA burden trends spanning 1990 to 2021. The global population is aging rapidly; a demographic transition strongly correlated with increased AA incidence and rupture risk. Simultaneously, global trends in key AA risk factors exhibit marked divergence: while smoking prevalence has declined in many high-income regions, it remains stubbornly high or is increasing in others, and hypertension control shows significant geographical disparities despite overall global increases in prevalence.^7,8^ The interplay of these demographic and risk factor dynamics (population aging, heterogeneous changes in smoking patterns, and uneven progress in hypertension management) is likely reshaping the global landscape of AA burden in complex ways that prior GBD analyses (based on pre-2020 data and less refined methods) cannot adequately capture. A systematic re-assessment is therefore essential to understand how these forces have influenced AA mortality and morbidity across different populations and settings.

 Consequently, this study leverages the enhanced GBD 2021 dataset to provide a timely and comprehensive update on the global AA burden. We move beyond simply stating the lack of recent studies to explicitly address critical public health knowledge gaps exposed by recent global events and methodological progress. Our specific, testable objectives are to: (1) quantify how AA-related mortality and DALYs have varied from 1990 to 2021, stratified by age, sex, and socio-demographic Index (SDI) quintile; and (2) determine the proportion of the AA burden attributable to key modifiable risk factors globally and across SDI strata.

## Materials and Methods

###  Data Sources

 This study utilized data from the GBD 2021 dataset, which included individuals with AA from 1990 to 2021 across all age groups.^9^ Consistent with the GBD methodology, our analysis focused on individuals aged ≥ 15 years due to: (1) the clinical rarity of AA in pediatric populations, as AA predominantly results from age-related degenerative processes and atherosclerosis, and (2) the absence of AA burden estimates for ages < 15 in the GBD 2021 dataset, reflecting both biological plausibility and data availability constraints. Data on AA-related deaths, DALYs, and their corresponding age-standardized rates (ASRs) were obtained via the Global Health Data Exchange (GHDx) platform (http://ghdx.healthdata.org/gbd-results-tool), along with 95% uncertainty intervals (UIs). The SDI quintile thresholds, consistent with prior GBD studies, represent standardized population divisions for global comparative analysis: low SDI (0‒0.45), low-middle SDI (0.45‒0.61), middle SDI (0.61‒0.69), high-middle SDI (0.69‒0.81) and high SDI (0.81‒1).^15^ Geographically, the globe was divided into 21 regions. ASRs were calculated using the GBD World Population Criteria, while crude rates were used for specific age groups (e.g. 15‒19 years, 20‒24 years, 25‒29 years, up to 95 + years), as these were the only rates provided by GBD 2021. The International Classification of Diseases, 10th Revision (ICD-10), codes corresponding to the GBD AA cause code (G174) include I71, I71.0, I71.1, I71.2, I71.3 I71.4, I71.5, I71.6, I71.8, I71.9. Data on the proportion of deaths and DALYs attributable to level 2 to 4 risk factors were accessed via the GBD Outcomes Tool (https://vizhub.healthdata.org/gbd-results/). Our risk factor analysis comprehensively evaluated smoking, hypertension, dietary components, and other metabolic determinants of AA burden. Detailed definitions of risk factor exposure metrics are available in the GBD 2021 Methods Appendices (available at: https://www.healthdata.org/gbd/methods-appendices-2021). Population attributable fractions (PAFs) were calculated to evaluate the impact of specific risk factors. Exposure data were modelled using either spatiotemporal Gaussian process regression or DisMod-MR 2.1, which are Bayesian statistical models developed over the past 12 years for GBD analyses, and methods for estimating their contributions to AA-related deaths have been previously published.^12,15,16^

 No ethical review was required for this study because data from the 2021 GBD are publicly available. This study adheres to the Guidelines for Accurate and Transparent Health Estimates Reporting (GATHER).^17^

###  Statistical Analysis

 This study employed ASR for deaths (ASDR), DALYs (ASDALYR), as well as estimated annual percentage change (EAPC), to quantify the global burden of AA.^18^ Standardization was necessary for comparing populations with different age structures or for analyzing temporal changes in the age structure of the same population. The ASRs were reported per 100 000 people, with ASDR and ASDALYR expressed accordingly. The EAPC involves a linear regression model y = α + βx + ε, where y = ln (ASR), x = calendar year and ε is the error. EAPC is then calculated as 100 × (exp (*β*)- 1) and reported with a 95% confidence interval (CI) to describe temporal trends. An increasing trend in ASR was identified if both the EAPC estimate and its lower 95% CI were greater than zero, whereas a declining trend was observed when both the estimate and its upper and lower 95% CI limits were below zero. If the 95% CI of the EAPC included zero, the ASR was considered stable. Spatial trends of EAPC for ASDR and ASDALYR were analyzed across 204 countries and territories from 1990 to 2021. Temporal trends in ASDR and ASDALYR for AA burden during the same period were assessed globally and across SDI levels. The relationship between SDI and AA burden, along with its spatial and temporal characteristics, was evaluated using Pearson’s correlation coefficient. Detailed descriptions of the analytical methods can be found in previous studies.^9,10^ Descriptive statistics were generated for all key variables, with results expressed as means and 95% UIs. For trend analyses, a *P* value < 0.05 was considered statistically significant. All statistical analyses and data visualizations were performed using R (version 4.2.3).

## Results

###  Deaths and DALYs of AA 

**Figure 1:**
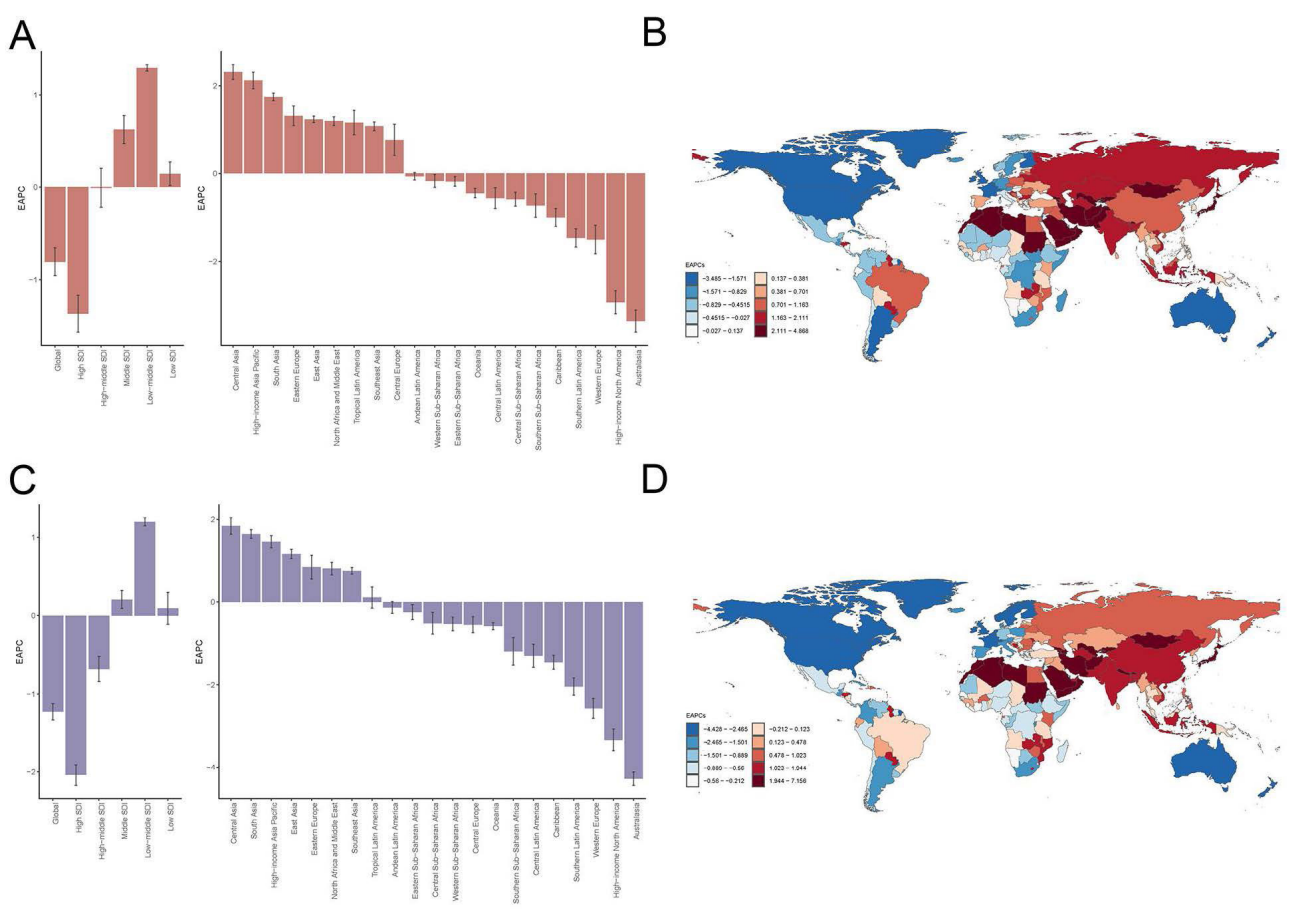
EAPC in ASDR and ASDALYR of Aortic Aneurysm. A. The EAPC of ASDR with 27 regions. B. The EAPC of ASDRs in 204 countries and territories between 1990 and 2021. C. The EAPC of ASDALYR in 27 regions. D. The EAPC of ASDALYR in 204 countries and territories between 1990 and 2021. EAPC, estimated annual percent-age change; ASDR, age standardized death rate; ASDALYR, age-standardized disability-adjusted life years rate; SDI, Sociodemographic Index.

 Globally, AA and dissection accounted for 153927 deaths (95% UI: 138413‒165739) in 2021, marking a 74.218% increase from 88353 deaths (95% UI: 83090‒93492) in 1990 (Table 1). Despite this increase in absolute deaths,the global ASDR decreased from 2.538/100 000 persons (95% UI: 2.347‒2.690) in 1990 to 1.861/100 000 persons (95% UI: 1.666‒2.003) in 2021. Over this period, the global trend in ASDR showed a decline, with an EAPC of -0.808 (95% CI: -0.958 to -0.657). Among the SDI regions, the high-SDI region reported the highest number of deaths in 2021 (67202, 95% UI: 57735‒72287) and the highest ASDR in both 1990 (4.763/100 000, 95% UI: 4.457‒4.912) and 2021 (2.866/100 000, 95% UI: 2.515‒3.057). However, this region exhibited a significant declining trend for ASDR, with an EAPC of -1.368 (95% CI: -1.567 to -1.170) over the past three decades (Figure 1A). At the regional level, the greatest number of AA deaths in 2021 occurred in South Asia (15979, 95% UI: 11379‒23410). The highest ASDR was recorded in High-income Asia Pacific (4.376, 95% CI:3.717‒4.753), while the lowest ASDR was observed in East Asia (0.502, 95% CI: 0.408-0.627) in 2021. From 1990 to 2021, the absolute number of AA deaths increased across almost all GBD regions, except for Australasia, Western Europe, and High-income North America. The most pronounced decrease in ASDR was observed in Australasia, with an EAPC of -3.350 (95% CI: -3.602 to -3.098), followed by High-income North America (-2.924, 95% CI: -3.189 to -2.659). Conversely, the greatest increase in ASDR occurred in Central Asia with an EAPC of 2.314 (95% CI: 2.147‒2.481), followed by High-income Asia Pacific (2.121, 95% CI:1.933‒2.310) from 1990 to 2021. On a country-level analysis, the top five countries with the highest positive EAPCs in ASDR were Georgia, Sultanate of Oman, Republic of Uzbekistan, Republic of Yemen, and Republic of Sudan (Figure 1B). In contrast, the fastest declines in ASDR were observed in Guam, Australia, Canada, Puerto Rico and the United States of America.

**Table 1:** Deaths of Aortic Aneurysm Between 1990 and 2021 at the Global and regional level

**Location**	**1990**		**2021**		**EAPC in ASDR** ** (95% CI)**
	**Deaths Cases (95% UI)**	**ASDR Per 100,000 (95% UI)**	**Deaths Cases (95% UI)**	**ASDR Per 100,000 (95% UI)**	
Global	88353 (83090, 93492)	2.538 (2.347, 2.690)	153927 (138413, 165739)	1.861 (1.666, 2.003)	-0.808 (-0.958, -0.657)
SDI					
Low SDI	2557 (1568, 4437)	1.366 (0.832, 2.371)	6371 (3932, 10434)	1.482 (0.914, 2.442)	0.143 (0.015, 0.270)
Low-middle SDI	4608 (3664, 6272)	0.888 (0.709, 1.197)	16808 (13956, 22468)	1.313 (1.089, 1.758)	1.292 (1.257, 1.327)
Middle SDI	8804 (8110, 9844)	1.026 (0.942, 1.139)	28528 (25797, 30959)	1.155 (1.045, 1.253)	0.627 (0.476, 0.778)
High-middle SDI	18321 (17508, 19197)	1.988 (1.883, 2.079)	34827 (32309, 37274)	1.788 (1.656, 1.915)	-0.008 (-0.218, 0.202)
High SDI	53929 (50582, 55553)	4.763 (4.457, 4.912)	67202 (57735, 72287)	2.866 (2.515, 3.057)	-1.368 (-1.567, -1.170)
Regions					
East Asia	2936 (2374, 3719)	0.359 (0.294, 0.443)	10199 (8229, 12817)	0.502 (0.408, 0.627)	1.241 (1.167, 1.315)
Southeast Asia	2069 (1670, 2586)	1.031 (0.827, 1.289)	7391 (6476, 8513)	1.388 (1.208, 1.604)	1.081 (0.984, 1.179)
Oceania	50 (38, 68)	2.291 (1.793, 2.977)	117 (91, 151)	1.942 (1.543, 2.452)	-0.448 (-0.552, -0.343)
Central Asia	430 (374, 513)	0.941 (0.814, 1.128)	1443 (1280, 1615)	1.977 (1.765, 2.210)	2.314 (2.147, 2.481)
Central Europe	4379 (4217, 4522)	3.073 (2.945, 3.179)	6682 (6141, 7318)	2.925 (2.686, 3.208)	0.771 (0.410, 1.133)
Eastern Europe	6812 (6583, 7085)	2.519 (2.427, 2.620)	13406 (12354, 14430)	3.825 (3.524, 4.115)	1.321 (1.100, 1.542)
High-income Asia Pacific	5277 (4879, 5532)	2.788 (2.555, 2.930)	25773 (20940, 28524)	4.376 (3.717, 4.753)	2.121 (1.933, 2.310)
Australasia	1902 (1788, 2012)	8.025 (7.498, 8.495)	1549 (1354, 1680)	2.599 (2.289, 2.809)	-3.350 (-3.602, -3.098)
Western Europe	29016 (27281, 29890)	4.780 (4.498, 4.921)	27511 (24098, 29189)	2.570 (2.304, 2.711)	-1.501 (-1.824, -1.177)
Southern Latin America	2121 (1978, 2296)	4.712 (4.404, 5.092)	2352 (2167, 2528)	2.635 (2.431, 2.834)	-1.465 (-1.669, -1.260)
High-income North America	19569 (18209, 20354)	5.296 (4.935, 5.502)	13970 (12471, 14793)	2.086 (1.887, 2.199)	-2.924 (-3.189, -2.659)
Caribbean	921 (851, 981)	3.790 (3.509, 4.038)	1405 (1240, 1583)	2.590 (2.287, 2.922)	-1.003 (-1.205, -0.801)
Andean Latin America	193 (164, 228)	1.005 (0.860, 1.191)	538 (449, 644)	0.935 (0.783, 1.119)	-0.066 (-0.150, 0.017)
Central Latin America	1198 (1150, 1240)	1.560 (1.485, 1.619)	3293 (2865, 3768)	1.368 (1.191, 1.563)	-0.561 (-0.798, -0.323)
Tropical Latin America	2901 (2780, 2994)	3.357 (3.191, 3.478)	10173 (9352, 10729)	4.038 (3.701, 4.259)	1.167 (0.891, 1.444)
North Africa and Middle East	1059 (776, 1456)	0.657 (0.490, 0.891)	3694 (3203, 4257)	0.893 (0.776, 1.028)	1.199 (1.102, 1.295)
South Asia	3457 (2168, 5573)	0.711 (0.451, 1.127)	15979 (11379, 23410)	1.220 (0.879, 1.778)	1.748 (1.660, 1.837)
Central Sub-Saharan Africa	476 (263, 788)	2.692 (1.484, 4.435)	1057 (591, 1706)	2.407 (1.347, 3.880)	-0.585 (-0.743, -0.426)
Eastern Sub-Saharan Africa	1135 (677, 1948)	1.817 (1.072, 3.051)	2636 (1427, 4293)	1.804 (0.986, 2.962)	-0.183 (-0.291, -0.075)
Southern Sub-Saharan Africa	737 (604, 862)	3.090 (2.485, 3.658)	1237 (1123, 1349)	2.455 (2.231, 2.677)	-0.731 (-0.997, -0.465)
Western Sub-Saharan Africa	1715 (905, 3053)	2.356 (1.247, 4.177)	3523 (1745, 5980)	2.186 (1.096, 3.678)	-0.171 (-0.316, -0.025)

SDI, socio-demographic index; ASDR, age-standardized death rates; UI, uncertainty interval; CI, confidence interval.

 Globally, the AA-related DALYs in 2021 were 3 107 762 (95% UI: 2 857 320‒3 353 858), representing a 64.944% increase from 1884127 (95% UI: 1 784 177‒2 006 704) in 1990 (Table 2). In contrast, the global ASDALYR decreased from 48.789/100,000 persons (95% UI: 46.009‒51.805) in 1990 to 36.543/100 000 persons (95% UI: 33.515‒39.455) in 2021, with an EAPC of -1.227 (95% CI: -1.331 to -1.124). In the SDI analysis, the high-SDI region consistently had the highest ASDALYR, declining from 92.052/100 000 persons (95% UI: 87.991‒94.226) in 1990 to 54.481/100 000 persons (95% UI: 50.258‒57.113) in 2021, with a significant EAPC of -2.040 (95% CI: -2.175 to -1.905) (Figure 1C). Conversely, the low-middle SDI region had the lowest ASDALYR in 1990 (18.846/100 000 persons, 95% UI: 14.980‒25.692) but showed a marked increase to 27.490/100 000 persons (95% UI: 22.934‒36.665) in 2021, with an EAPC of 1.196 (95% CI: 1.145‒1.247). Regionally, the largest number of DALYs was found in Western Europe (449 312, 95% UI: 408 554‒471 934), followed by South Asia in 2021. The highest ASDALYR was observed in Eastern Europe (91.309/100 000 persons, 95% UI: 83.882‒98.462) and the lowest was observed in East Asia (13.313/100 000 persons, 95% UI: 10.595‒16.969). The fastest decline in ASDALYR was recorded in Australasia, with an EAPC of -4.284 (95% CI: -4.447 to -4.122), followed by High-income North America, Western Europe and Southern Latin America. In contrast, the fastest increase was observed in Central Asia, with an EAPC of 1.834 (95% CI: 1.635‒2.034), followed by South Asia, High-income Asia Pacific and East Asia. At the country level, the highest EAPCs in ASDALYR were found in Georgia, Republic of Uzbekistan, and Islamic Republic of Afghanistan, while the lowest EAPCs were observed in Australia, Canada and United Kingdom (Figure 1D).

**Table 2:** Aortic Aneurysm Disability-Adjusted Life Years Between 1990 and 2021 at the Global and Regional Level

**Regions**	**1990**		**2021**		**EAPC in ASDALYR** ** (95 % CI)**
	**DALYs Cases (95% UI)**	**ASDALYR Per 100,000 (95% UI)**	**DALYs Cases (95% UI)**	**ASDALYR Per 100,000 (95% UI)**	
Global	1884127 (1784177, 2006704)	48.789 (46.009, 51.805)	3107762 (2857320, 3353858)	36.543 (33.515, 39.455)	-1.227 (-1.331, -1.124)
SDI					
Low SDI	65889 (41122, 114005)	28.730 (17.674, 49.857)	162855 (99208, 267958)	30.812 (18.937, 50.420)	0.093 (-0.111, 0.297)
Low-middle SDI	116754 (92573, 160125)	18.846 (14.980, 25.692)	398280 (333990, 529921)	27.490 (22.934, 36.665)	1.196 (1.145, 1.247)
Middle SDI	231091 (212403, 260257)	21.645 (19.925, 24.249)	662164 (600453, 722827)	24.882 (22.557, 27.102)	0.205 (0.090, 0.321)
High-middle SDI	439742 (421449, 464651)	44.020 (42.182, 46.414)	760354 (709824, 817072)	39.687 (37.020, 42.656)	-0.683 (-0.843, -0.523)
High SDI	1027749 (982266, 1051562)	92.052 (87.991, 94.226)	1120317 (1010907, 1182280)	54.481 (50.258, 57.113)	-2.040 (-2.175, -1.905)
Regions					
East Asia	94823 (75786, 122685)	9.505 (7.659, 12.133)	269443 (213791, 344576)	13.313 (10.595, 16.969)	1.160 (1.049, 1.271)
Southeast Asia	48941 (39584, 61285)	19.573 (15.764, 24.401)	158458 (137445, 182389)	25.868 (22.552, 29.830)	0.751 (0.674, 0.829)
Oceania	1422 (1056, 1966)	47.297 (36.362, 63.947)	3301 (2496, 4382)	41.501 (32.091, 53.542)	-0.584 (-0.668, -0.500)
Central Asia	11701 (10365, 13841)	23.597 (20.778, 27.999)	35160 (30895, 39777)	42.684 (37.784, 47.927)	1.834 (1.635, 2.034)
Central Europe	101856 (98807, 104955)	69.082 (66.844, 71.148)	134479 (123463, 148457)	63.672 (58.323, 70.612)	-0.549 (-0.743, -0.355)
Eastern Europe	171620 (166524, 177811)	62.244 (60.342, 64.472)	304641 (280046, 328883)	91.309 (83.882, 98.462)	0.842 (0.555, 1.129)
High-income Asia Pacific	104846 (98300, 110358)	52.795 (49.285, 55.556)	378280 (325387, 408692)	79.732 (71.778, 84.483)	1.456 (1.309, 1.602)
Australasia	35502 (33653, 37409)	148.048 (140.294, 155.961)	24880 (22367, 26611)	45.525 (41.268, 48.612)	-4.284 (-4.447, -4.122)
Western Europe	538001 (515288, 551079)	92.416 (88.689, 94.607)	449312 (408554, 471934)	48.514 (45.110, 50.595)	-2.570 (-2.810, -2.330)
Southern Latin America	46977 (43919, 50952)	101.001 (94.448, 109.407)	48689 (45138, 52222)	56.505 (52.391, 60.580)	-2.043 (-2.252, -1.833)
High-income North America	375208 (356860, 385918)	105.196 (100.364, 108.037)	271835 (253830, 283631)	45.431 (42.908, 47.247)	-3.334 (-3.597, -3.069)
Caribbean	18250 (17039, 19521)	70.712 (66.014, 75.455)	26859 (23699, 30389)	49.954 (44.084, 56.537)	-1.457 (-1.624, -1.291)
Andean Latin America	4767 (3966, 5655)	21.626 (18.126, 25.673)	12092 (10091, 14633)	20.067 (16.764, 24.271)	-0.134 (-0.278, 0.011)
Central Latin America	30018 (29077, 31001)	33.864 (32.705, 35.028)	72062 (62327, 83068)	28.768 (24.925, 33.114)	-1.299 (-1.578, -1.020)
Tropical Latin America	78229 (75914, 80470)	79.835 (76.923, 82.314)	235449 (221294, 246968)	91.101 (85.463, 95.499)	0.108 (-0.149, 0.365)
North Africa and Middle East	31346 (22507, 43520)	16.347 (11.896, 22.520)	96331 (83716, 112193)	19.959 (17.329, 23.115)	0.806 (0.655, 0.957)
South Asia	89111 (54740, 145521)	15.282 (9.538, 24.678)	367475 (260596, 539642)	24.998 (17.792, 36.645)	1.642 (1.538, 1.746)
Central Sub-Saharan Africa	12490 (6935, 20437)	56.258 (31.180, 92.284)	28159 (15580, 46218)	50.549 (28.248, 81.641)	-0.515 (-0.778, -0.252)
Eastern Sub-Saharan Africa	30462 (18603, 52523)	38.507 (22.978, 66.059)	72025 (38938, 115788)	38.527 (20.814, 62.597)	-0.247 (-0.426, -0.067)
Southern Sub-Saharan Africa	18239 (15600, 20674)	63.544 (52.897, 73.439)	30967 (27977, 34412)	51.989 (46.991, 57.205)	-1.195 (-1.524, -0.864)
Western Sub-Saharan Africa	40319 (21112, 72610)	46.437 (24.328, 83.092)	87865 (42722, 151832)	43.504 (21.402, 74.120)	-0.532 (-0.694, -0.369)

SDI, socio-demographic index; UI, uncertainty interval; EAPC, Estimated annual percentage change; DALYs, disability-adjusted life years; ASDALYR, age-standardized DALY rate.

###  Gender and Age Distribution of AA Burden

**Figure 2:**
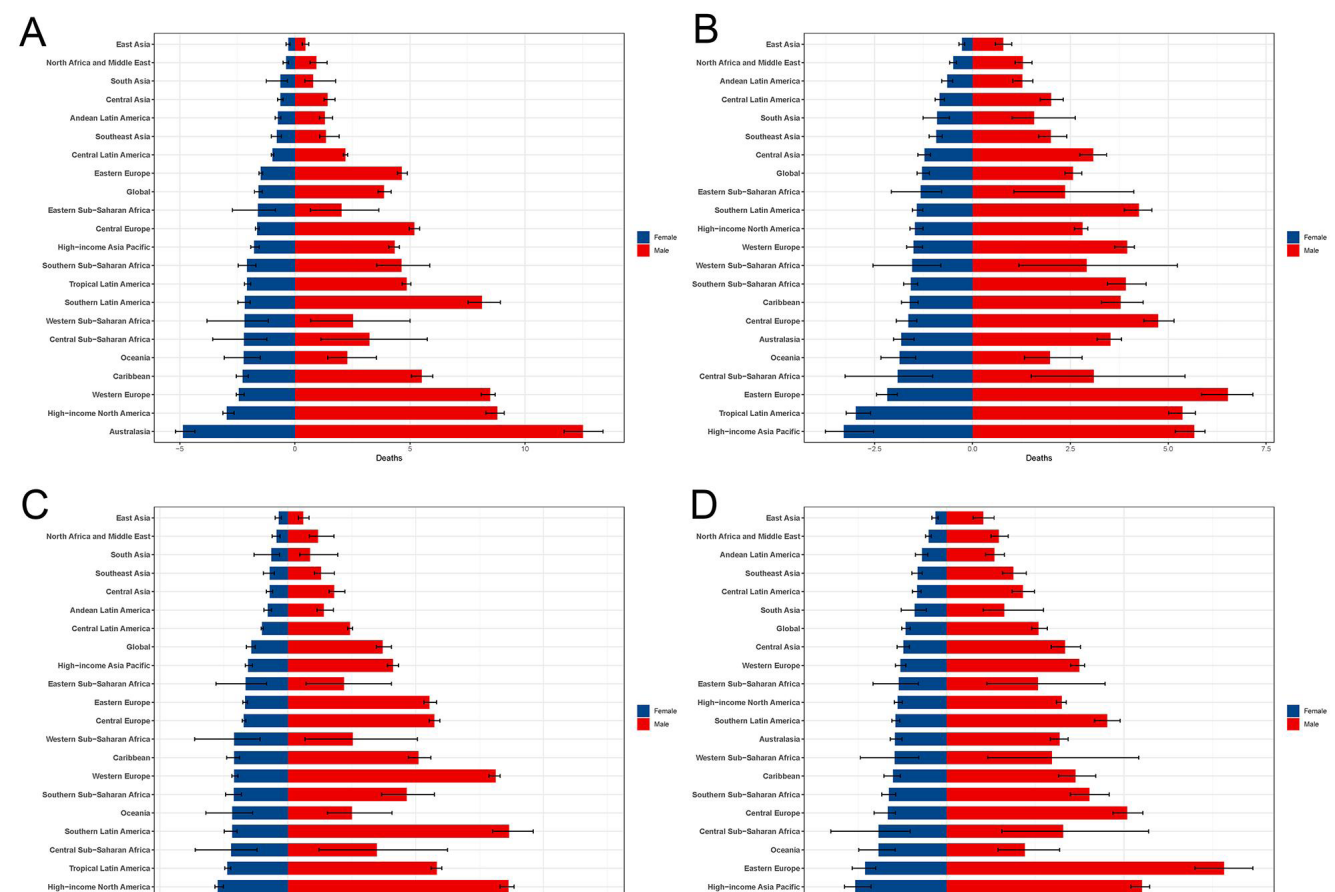
Difference in ASDR and ASDALYR by 21 GBD Regions and Gender in 1990 and 2021. A. ASDRs in 21 regions in 1990. B. ASDRs in 21 regions in 2021. C. ASDALYR in 21 regions in 1990. D. ASDALYR in 21 regions in 2021. ASDR, age standardized death rate; ASDALYR, age-standardized disability-adjusted life years rate; GBD, Global Burden of Disease.

**Figure 3:**
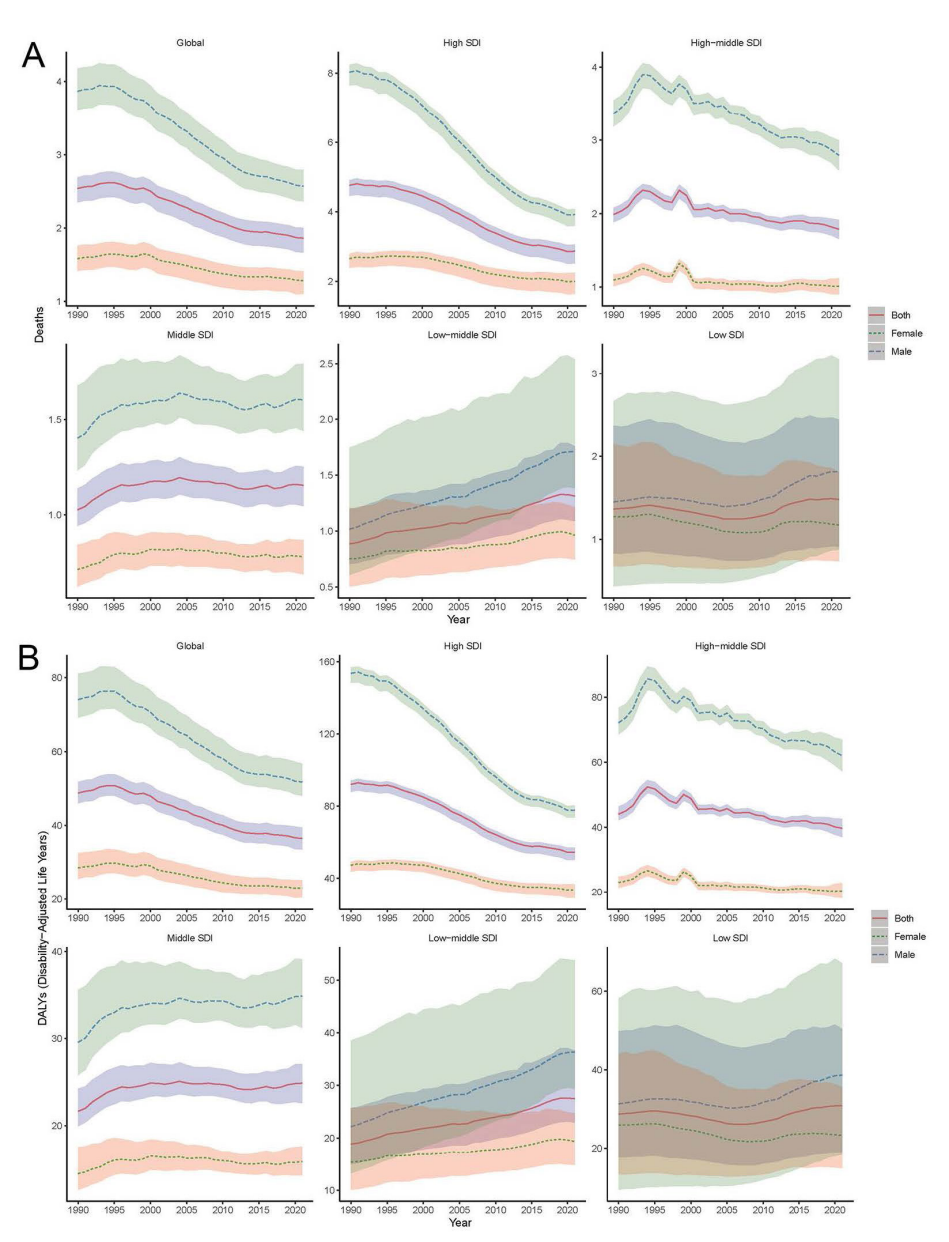
Temporal trends of ASDR (A) and ASDALYR (B) of Aortic Aneurysm, globally and by sociodemographic index (five categories; countries with a high, high-middle, middle, low-middle, or low sociodemographic index) from 1990 to 2021. ASDR, age standardized death rate; ASDALYR, age-standardized disability-adjusted life years rate; SDI, Sociodemographic Index.

 Globally, both the ASDR and ASDALYR were consistently lower in females than in males across the 21 GBD regions in 1990 and 2021 (Figures 2 and 3). The lowest ASDR and ASDALYR were recorded in East Asia in both years. In 1990, the highest ASDR of AA was found in Australasia (8.025/100 000 persons, 95% UI:7.498‒8.495) but shifted to high-income Asia Pacific in 2021 (4.376/100 000 persons, 95% UI: 3.717‒4.753) (Table 1 and Figures 2A and 2B). Similarly, the highest ASDALYR was observed in Australasia (148.048/100 000 persons, 95% UI:140.294‒155.961) in 1990 but transitioned to Eastern Europe (91.309/100 000 persons, 95% UI: 83.882‒98.462) in 2021 (Table 2 and Figures 2C and 2D). Both ASDR and ASDALYR exhibited declining trends globally for both genders over the past 32 years (Figure 3). Analysis by SDI levels revealed that ASDR and ASDALYR increased with rising social development. However, low and low-middle SDI regions experienced the most rapid increases over time. In contrast, middle SDI regions showed relatively stable trends, while high and high-middle SDI regions demonstrated consistent declines.

**Figure 4:**
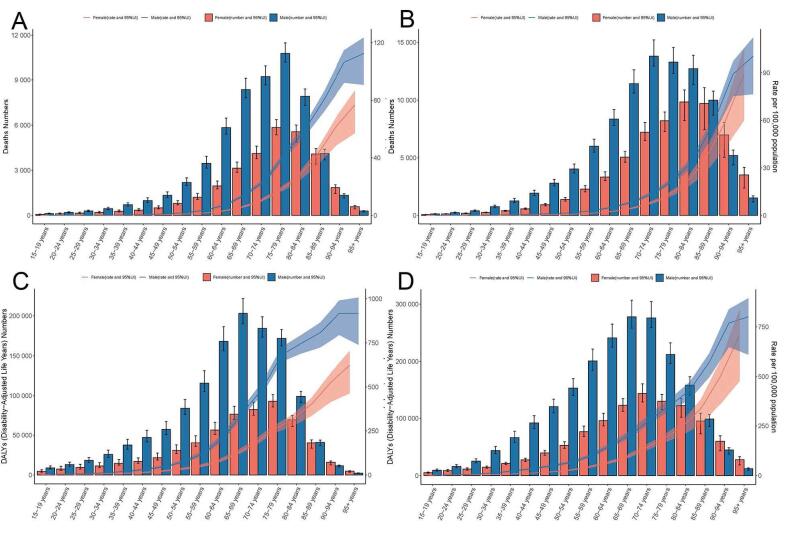
Age-Specific Burden of Aortic Aneurysm in Males and Females. A. deaths and ASDR in 1990. B. deaths and ASDR in 2021. C. DALYs and ASDALYR in 1990. D. DALYs and ASDALYR in 2021. UI, uncertainty interval; ASDR, age standardized death rate; ASDALYR, age-standardized disability-adjusted life years rate.

 In terms of absolute numbers, males accounted for a greater number of deaths and DALYs compared to females across all age groups from 1990 to 2021 (Figure 4). In 1990, the highest number of deaths occurred among individuals aged 75‒79 years for both genders. By 2021, the peak shifted to males aged 70‒74 years and females aged 80-84 years. For DALYs, the highest burden in 1990 was seen in males aged 65‒69 years and females aged 75‒79 years. By 2021, the peak shifted to males aged 65‒69 years and females aged 70‒74 years. Globally, both ASDR and ASDALYR showed an increasing trend with age, with the highest rates consistently observed in the oldest age group ( ≥ 95 years) for both genders.

###  Correlation Between AA Burden and SDI

**Figure 5:**
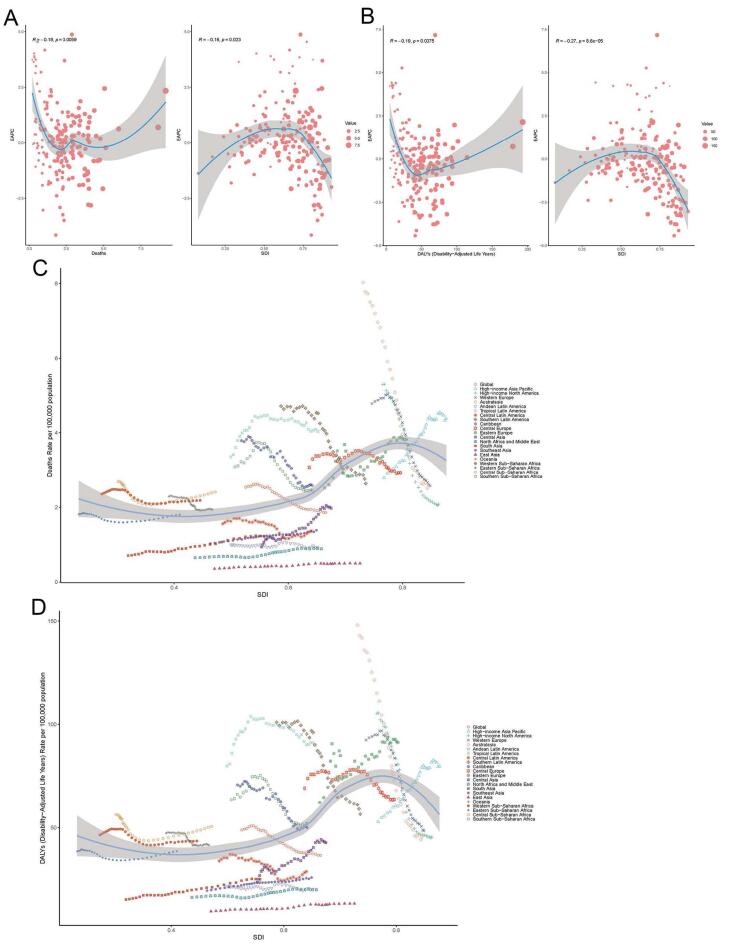
Correlation Between EAPC and SDI. A. Correlation between EAPC and ASDR, and SDI of 204 countries and territories in 2021. B. Correlation between EAPC and ASDALYR, and SDI of 204 countries and territories in 2021. C. Correlation between ASDR, and SDI of 21 regions from 1990 to 2021. D. Correlation between ASDALYR and SDI of 21 regions from 1990 to 2021. The R indices and *P* values presented were derived from Pearson correlation analysis.

 EAPC was negatively correlated with the ASDR (ρ = - 0.19, *P* = 0.0059) and the SDI of 204 countries and territories in 2021 (Figure 5A). The EAPC of ASDR displayed an inverted U-shaped relationship across SDI regions, with middle SDI regions showing higher EAPC values than low or high SDI regions (ρ = - 0.16, *P* = 0.023). Countries with higher SDI levels experienced more pronounced decreases in ASDR for AA. The relationship between the EAPC of ASDALYR (ρ = - 0.19, *P* = 0.0075) and the SDI (ρ = - 0.27, *P* < 0.001) mirrored the same pattern of the EAPC of deaths (Figure 5B).

 Global and regional trends in ASDR (ρ = 0.487, *P* < 0.001) and ASDALYR (ρ = 0.466, *P* < 0.001) in relation to SDI were expressed in the annual time series from 1990 to 2021 (Figures 5C and 5D). Regions, especially those with low SDI, generally followed the trend of increased ASDR and ASDALYR as SDI improved. However, regions with an SDI greater than 0.75 showed a downward trend in both ASDR and ASDALYR during the study period. At the global level, ASDR and ASDALYR have consistently declined with increasing SDI values over the past 32 years, reflecting improvements in healthcare access, prevention strategies, and disease management in higher-SDI regions.

###  Attributable Risk Factors for AA Burden

**Figure 6:**
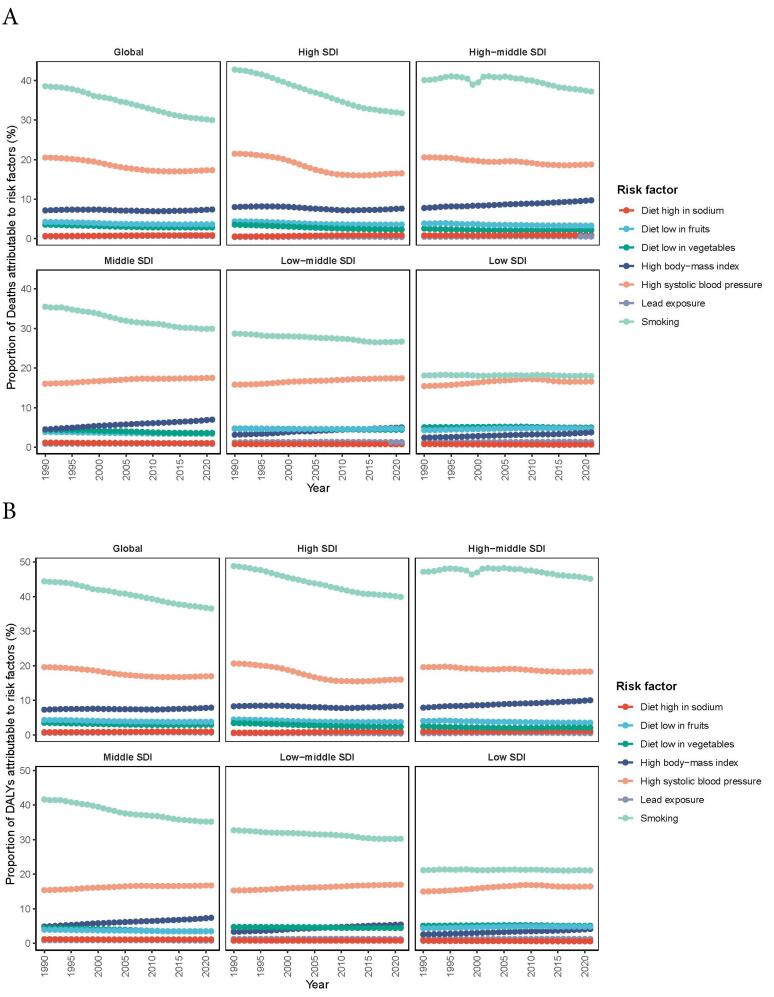
AA deaths and DALYs attributable to risk factors. A. Proportion of AA deaths attributable to risk factors in global and 5 SDI quintiles regions, from 1990 to 2021. B. Proportion of AA DALYs attributable to risk factors in global and 5 SDI quintiles regions, from 1990 to 2021.

 To further explore the composition of AA burden, attributable risk factors were analyzed, and diets high in sodium, diets low in fruits, diets low in vegetables, lead exposure, high body-mass index, high systolic blood pressure and smoking, were screened out in the risk factor hierarchy (Figures 6A and 6B). Smoking was identified as the most significant attributable risk factor, particularly in high/middle SDI regions. The contribution of smoking was far exceeding that of other risk factors from 1990 to 2021, followed by high systolic blood pressure. High systolic blood pressure maintained stable contributions overall, showing moderate increases in low-SDI areas but gradual declines in high-SDI regions. High body-mass index demonstrated diverging trends, with a substantial growth in middle-SDI regions contrasting with stable trends in high-SDI areas. Conversely, the proportions of AA burden attributable to dietary factors and lead exposure were much smaller, fluctuating across all SDI regions.

## Discussion

 This study comprehensively analyzed the global, regional, and national trends in deaths, DALYs, and risk factors associated with AA burden from 1990 to 2021, based on the GBD 2021 study. Our findings revealed a progressive increase in the absolute number of AA deaths and DALYs, alongside a declining trend in ASDR and ASDALYR. Males exhibited a greater number of deaths and DALYs than females across all age groups and GBD regions during the study period. Smoking was identified as the leading attributable risk factor for AA, despite its overall decline. These results highlight the persistent global burden of AA, emphasizing the need for targeted preventive strategies and resource allocation.

 Consistent with earlier GBD-based epidemiological studies, such as GBD 2017 and GBD 2019, the absolute number of AA deaths and DALYs increased from 1990 to 2021.^11,12^ However, ASDR and ASDALYR demonstrated a global downward trend, particularly in high-SDI regions, likely due to improved healthcare access and quality (HAQ index) and advancements in diagnostic and therapeutic technologies.^19^ Regional variations were noted in AA burden. Previously, the highest mean AA mortality and years of life lost (YLLs) rates of GBD regions were reported to be Australasia and Western Europe in 2010.^11^ The highest ASDR was observed in tropical Latin America and the lowest ASDR was observed in East Asia in both 2017 and 2019. The highest ASDALYR was observed in Oceania in 2017, and the lowest was observed in East Asia in 2017.^12,20^ Our results demonstrated that the highest ASDR and ASDALYR were observed in High-income Asia Pacific and Eastern Europe, respectively, and the lowest ASDR and ASDALYR were also observed in East Asia in 2021. The most pronounced decrease in ASDR was observed in Australasia, whereas the highest increase was in Central Asia from 1990 to 2021. The fastest decline of ASDALYR was found in Australasia, and Central Asia showed the fastest increase. Geographical analysis showed that Georgia was the country with the highest positive age-standardized annualized rates of change (ARC) mortality due to AA, while Australia was the one with the highest declining ARC. Our results on observation from the 204 countries and territories in these 31 years showed that the top positive EAPCs of ASDR were found in Georgia, while the country with the fastest decline of ASDR was found to be Guam, followed by Australia. These geographical disparities may stem from differences in healthcare infrastructure, diagnostic practices, and public health policies.^19^

 The burden of AA was inversely correlated with SDI, showing an inverted U-shaped relationship.^20,21^ A positive association was noted at the beginning while negative associations were observed when the SDI exceeded about 0.75. High-SDI regions, characterized by better medical resources and health management systems, experienced greater declines in AA burden, potentially due to aging populations, extended lifespans, and improved diagnostic technologies.^21,22^ Conversely, low income and low educational levels are associated with increased risk of abdominal AA rupture.^19,23^ Conversely, low-SDI regions demonstrated relatively smaller contributions to the global AA burden, partly due to limited diagnostic capabilities and underreporting. These findings underscore the need for equitable healthcare interventions and resources tailored to low-SDI regions.

 The AA burden increased with age, predominantly affecting individuals aged over 55 years, which explains the high burden in high-SDI regions with aging populations. Besides, the peak age for AA burden was earlier in males than females. The highest number of deaths occurred at ages 70‒74 years among males and 80‒84 years among females, and the number of DALYs was highest among males aged 65‒69 years and females aged 70‒74 years in 2021. Males were also reported to have higher ASDR and ASDALYR than females globally and regionally, consistent with previous research findings.^19^ These discrepancies may be attributable to the difference in smoking between genders, which was recognized as the major risk factor in our study. Notably, males experienced a more pronounced decline in ASDR and ASDALYR than females globally and in high and high-middle regions, highlighting the need for gender-specific interventions.^20^

 We calculated the PAFs of diets high in sodium, diets low in fruits, diets low in vegetables, lead exposure, high body-mass index, high systolic blood pressure and smoking to quantify the effect. We found smoking to be the dominant contributor but tended to decrease, which could be considered an important reason for the decline in AA burden and confirmed by the substantial decrease in the PAF of smoking in high, high-middle and middle SDI regions observed over the past 31 years in our study.^24^ Reduction in tobacco consumption was observed to be related to a decline in incidence of abdominal AA in developed countries.^18^ A dose-response relationship between smoking and AA deaths was also found in a prospective study.^25^ These findings should compel governments to further strengthen tobacco control measures for limiting AA burden at the societal level. Despite global declines in age-standardized AA rates from 1990‒2021, significant disparities persist, with males experiencing higher mortality than females, and Central Asia showing rising burdens. Our epidemiological findings underscore the necessity for geographically tailored prevention approaches, prioritizing evidence-based tobacco control interventions (e.g. WHO Framework Convention-compliant taxation) in high-prevalence regions, complemented by targeted screening programs in areas demonstrating significant temporal increases in ASRs. Based on the GBD 2019 study, smoking remains the predominant risk factor for men while hypertension has been the predominant risk factor for women; it is also predicted that hypertension would surpass smoking as the top risk factor.^13^ The GBD 2019 projection was based on risk factor trajectory modeling, whereas our 2021 findings represent actual epidemiological measurements. The anticipated transition may require longer observation periods, particularly given the persistent smoking prevalence in certain female cohorts and time lags in hypertension detection and reporting. While our study confirmed smoking’s predominance overall, we acknowledge that our sex-stratified risk factor analysis was not sufficiently granular to examine age-specific risk factor transitions. High sodium intake has been linked to hypertension and we could see that there has been impressive effects on reducing dietary salt consumption and blood pressure control since these two risk factors only present much smaller PAFs compared to smoking in our study.

## Conclusion

 This is the first study to utilize GBD 1990‒2021 data for a comprehensive global analysis of AA deaths, DALYs, and risk factors. Limitations common to the GBD study are also applicable to this study.^26,27^ Inability to obtain individual data prevents analyzing the impact of different interventions on mortality. Lack of subtype classification prevents analysis of how thoracic versus abdominal AA may differentially contribute to regional patterns - an important area for future research with more granular data. The number of AA patients in low and low-middle SDI regions is relatively small, which may still underestimate AA deaths due to competing mortality (e.g. infectious diseases) and diagnostic gaps. Despite these limitations, the GBD study’s robust calibration models minimize the impact of these issues, ensuring reliable results.^26^ From 1990 to 2021, declining trends were observed in the ASRs of AA-related deaths and DALYs, though significant geographic disparities persisted, with Central Asia and high-income Asia Pacific experiencing rising burdens. Notably, despite employing GBD’s robust covariate adjustment framework, our findings remain susceptible to residual confounding from unmeasured variables, particularly genetic factors known to influence AA pathogenesis, which may partially explain the observed epidemiological patterns independent of prevention measures. The AA burden remained consistently higher in males than females across all regions, particularly peaking at ages 70‒74 for deaths. While multiple factors contributed to these patterns, smoking was identified as the most significant modifiable risk factor. These findings provide the latest insights and a comprehensive overview of AA burden, serving as a foundation for designing targeted preventive strategies and enhancing vascular health globally.

## References

[1] Bossone E, Eagle KA (2021). Epidemiology and management of aortic disease: aortic aneurysms and acute aortic syndromes. Nat Rev Cardiol.

[2] Roth GA, Johnson C, Abajobir A, Abd-Allah F, Abera SF, Abyu G (2017). Global, regional, and national burden of cardiovascular diseases for 10 causes, 1990 to 2015. J Am Coll Cardiol.

[3] Hu B, Feng J, Wang Y, Hou L, Fan Y (2024). Transnational inequities in cardiovascular diseases from 1990 to 2019: exploration based on the Global Burden of Disease Study 2019. Front Public Health.

[4] Kuzmik GA, Sang AX, Elefteriades JA (2012). Natural history of thoracic aortic aneurysms. J Vasc Surg.

[5] Erbel R, Aboyans V, Boileau C, Bossone E, Bartolomeo RD, Eggebrecht H (2014). 2014 ESC Guidelines on the diagnosis and treatment of aortic diseases: document covering acute and chronic aortic diseases of the thoracic and abdominal aorta of the adult The Task Force for the Diagnosis and Treatment of Aortic Diseases of the European Society of Cardiology (ESC). Eur Heart J.

[6] Heikkinen M, Salenius JP, Auvinen O (2002). Ruptured abdominal aortic aneurysm in a well-defined geographic area. J Vasc Surg.

[7] Lim SS, Vos T, Flaxman AD, Danaei G, Shibuya K, Adair-Rohani H (2012). A comparative risk assessment of burden of disease and injury attributable to 67 risk factors and risk factor clusters in 21 regions, 1990-2010: a systematic analysis for the Global Burden of Disease Study 2010. Lancet.

[8] Ramanath VS, Oh JK, Sundt TM 3rd, Eagle KA (2009). Acute aortic syndromes and thoracic aortic aneurysm. Mayo Clin Proc.

[9] GBD 2021 Causes of Death Collaborators (2024). Global burden of 288 causes of death and life expectancy decomposition in 204 countries and territories and 811 subnational locations, 1990-2021: a systematic analysis for the Global Burden of Disease Study 2021. Lancet.

[10] GBD 2021 Diseases and Injuries Collaborators (2024). Global incidence, prevalence, years lived with disability (YLDs), disability-adjusted life-years (DALYs), and healthy life expectancy (HALE) for 371 diseases and injuries in 204 countries and territories and 811 subnational locations, 1990-2021: a systematic analysis for the Global Burden of Disease Study 2021. Lancet.

[11] Sampson UK, Norman PE, Fowkes FG, Aboyans V, Yanna S, Harrell FE Jr, et al. Global and regional burden of aortic dissection and aneurysms: mortality trends in 21 world regions, 1990 to 2010. Glob Heart 2014;9(1):171-80.e10. doi: 10.1016/j.gheart.2013.12.010. 25432126

[12] Wang Z, You Y, Yin Z, Bao Q, Lei S, Yu J (2022). Burden of aortic aneurysm and its attributable risk factors from 1990 to 2019: an analysis of the Global Burden of Disease Study 2019. Front Cardiovasc Med.

[13] Huang X, Wang Z, Shen Z, Lei F, Liu YM, Chen Z (2022). Projection of global burden and risk factors for aortic aneurysm - timely warning for greater emphasis on managing blood pressure. Ann Med.

[14] Wang H, Yu X, Guo J, Ma S, Liu Y, Hu Y (2024). Burden of cardiovascular disease among the Western Pacific region and its association with human resources for health, 1990-2021: a systematic analysis of the Global Burden of Disease Study 2021. Lancet Reg Health West Pac.

[15] Hong C, Liu Z, Gao L, Jin Y, Shi J, Liang R (2022). Global trends and regional differences in the burden of anxiety disorders and major depressive disorder attributed to bullying victimisation in 204 countries and territories, 1999-2019: an analysis of the Global Burden of Disease Study. Epidemiol Psychiatr Sci.

[16] Dai H, Zhang Q, Much AA, Maor E, Segev A, Beinart R (2021). Global, regional, and national prevalence, incidence, mortality, and risk factors for atrial fibrillation, 1990-2017: results from the Global Burden of Disease Study 2017. Eur Heart J Qual Care Clin Outcomes.

[17] Stevens GA, Alkema L, Black RE, Boerma JT, Collins GS, Ezzati M (2016). Guidelines for accurate and transparent health estimates reporting: the GATHER statement. Lancet.

[18] Laroche JP, Becker F, Baud JM, Miserey G, Jaussent A, Picot MC (2015). [Ultrasound screening of abdominal aortic aneurysm: lessons from Vesale 2013]. J Mal Vasc.

[19] Tyrovolas S, Tyrovola D, Giné-Vázquez I, Koyanagi A, Bernabe-Ortiz A, Rodriguez-Artalejo F (2022). Global, regional, and national burden of aortic aneurysm, 1990-2017: a systematic analysis of the Global Burden of Disease Study 2017. Eur J Prev Cardiol.

[20] Wei L, Bu X, Wang X, Liu J, Ma A, Wang T (2021). Global burden of aortic aneurysm and attributable risk factors from 1990 to 2017. Glob Heart.

[21] Zhang Y, Lai J (2024). Spatiotemporal trends in the burden of aortic aneurysms caused by high sodium intake from 1990 to 2019: a global, regional, and national analysis. Nutr Metab Cardiovasc Dis.

[22] Leong DP, Joseph PG, McKee M, Anand SS, Teo KK, Schwalm JD (2017). Reducing the global burden of cardiovascular disease, part 2: prevention and treatment of cardiovascular disease. Circ Res.

[23] Agyemang C, van den Born BJ (2018). Limited access to CVD medicines in low-income and middle-income countries: poverty is at the heart of the matter. Lancet Glob Health.

[24] Sidloff D, Stather P, Dattani N, Bown M, Thompson J, Sayers R (2014). Aneurysm global epidemiology study: public health measures can further reduce abdominal aortic aneurysm mortality. Circulation.

[25] Forsdahl SH, Singh K, Solberg S, Jacobsen BK (2009). Risk factors for abdominal aortic aneurysms: a 7-year prospective study: the Tromsø Study, 1994-2001. Circulation.

[26] Roth GA, Mensah GA, Johnson CO, Addolorato G, Ammirati E, Baddour LM (2020). Global burden of cardiovascular diseases and risk factors, 1990-2019: update from the GBD 2019 study. J Am Coll Cardiol.

[27] GBD 2019 Diseases and Injuries Collaborators (2020). Global burden of 369 diseases and injuries in 204 countries and territories, 1990-2019: a systematic analysis for the Global Burden of Disease Study 2019. Lancet.

